# Supplementary material for: Microreserves are an important tool for amphibian conservation
Source: Commun Biol. 2024 Sep 19;7:1177. doi: 10.1038/s42003-024-06510-0 (PMC11413221; doi:10.1038/s42003-024-06510-0)
Supplement: Supplementary file 2 — Supplementary Information [file 42003_2024_6510_MOESM2_ESM.docx]

**Supplementary Information**

**Microreservese are an important tool for amphibian conservation**

Emma Steigerwald^1,2*^, Julia Chen^1,2+^, Julianne Oshiro^1,2+^, Vance T. Vredenburg^1,3*^, Alessandro Catenazzi^4,5^, Michelle S. Koo^1*^

^1^ Museum of Vertebrate Zoology, University of California, Berkeley, Berkeley, CA, USA 94720

^2^ Department of Environmental Science, Policy, and Management, University of California, Berkeley, Berkeley, CA, USA 94720

^3^ Department of Biology, San Francisco State University, San Francisco, CA, USA, 94132

^4^ Department of Biological Sciences, Florida International University, Miami, FL 33199, USA

^5^ Centro de Ornitología y Biodiversidad, Lima, Peru

*Emma Steigerwald, Vance Vredenburg, Michelle Koo

**Table of Contents:**

**Supp. Table 1. Tests for distributional range difference between taxa.………………....** Page 1

**Supp. Table 2. GLMs for amphibian threatenedness responding to PA characteristics.** Page 2

**Supp. Table 3. GLMS for amphibian richness responding to PA characteristics…...…** Page 3

**Supp. Figure 1. Country profile plots………………….……………………………..……** Page 4

**Supp. Figure 2. Mexico: a case study…………………………….…………………..……** Page 4

**Supplementary Table 1.** Tests for distributional range difference between taxa, considering all species (above) and only species designated as IUCN threatened (below). P-values are marked as follows: <0.001, ***; <0.01, **; and <0.05, *. The total number of threatened and extinct species for which data was available in each taxonomic group was n_birds_=10,487; n_amphibians_=7,123; n_reptiles_=10,811; n_mammals_=5,850. The number of threatened and extinct species for which data was available in each taxonomic group was n_birds_=3,935; n_amphibians_=4,585, n_reptiles_=3,819, n_mammals_=3,752.

| **IUCN statuses included** | **Results of Kruskal-Wallis rank sum test** | **Results of pairwise Wilcoxon rank sum test with continuity correction (Bonferroni-adjusted p-values)** |
| --- | --- | --- |
| All species | chi-squared 5266.8  df 3  p-value 2.2·10^-16^*** | birds mammals reptiles  mammals < 2.0·10^-16^*** - -  reptiles <2.0·10^-16^*** <2.0·10^-16^*** -  amphibians <2.0·10^-16^*** <2.0·10^-16^*** <2.0·10^-16^*** |
| Threatened categories only (VU, EN, CR, EW, and EX) | chi-squared 687.81  df 3  p-value 2.2·10^-16^*** | birds mammals reptiles  mammals 0.009** - -  reptiles <2.0·10^-16^*** <2.0·10^-16^*** -  amphibians <2.0·10^-16^*** <2.0·10^-16^*** 0.018* |

**Supplementary Table 2.** Binomial generalized linear models examining how the proportion of amphibian species in a protected area that are threatened responds to its characteristics: its natural log-transformed area, its year of establishment, its centroid latitude, its IUCN protected area management category, or the interaction between its year of establishment and its natural log-transformed area. Model statistics are only included for the top three models by AIC. P-values are marked as follows: <0.001, ***; <0.01, **; and <0.05, *. This analysis includes 109,888 protected areas.

| **AIC** | **Variables** | **Coefficients**  **Estimate Std. error z-value p-value** | **Deviance (degrees of freedom)** | **McFadden's pseudo-r^2^** |  |
| --- | --- | --- | --- | --- | --- |
| 4519.8 | (proportion threatenedness) ~ latitude:ln(area) + latitude | intercept -1.53 0.05 -33.08 <2.0·10^-16^***  latitude -0.07 1.36·10^-3^ -50.25 <2.0·10^-16^***  lat:ln(area) 8.84·10^-4^ 2.54·10^-4^ 3.47 <5.2·10^-4^*** | Null: 8,505.9  (111,806)  Residual: 5,947.4 (111,804) | 0.035 |  |
| 4522.7 | (proportion threatenedness) ~ latitude | intercept -1.49 0.05 -33.29 <2.0·10^-16^***  latitude -0.07 1.3·10^-3^ -52.66 <2.0·10^-16^*** | Null: 8505.9 (111806)  Residual: 5959.4 (111805) | 0.034 |  |
| 4538.6 | (proportion threatenedness) ~ latitude + ln(area) | intercept -1.32 0.05 -26.10 <2.0·10^-16^***  latitude -0.07 1.4·10^-3^ -50.54 <2.0·10^-16^***  ln(area) -0.05 7.7·10^-3^ -6.84 <7.9·10^-12^*** | Null: 8505.9 (111806)  Residual: 5912.6 (111804) | 0.031 |  |
| 4551.0 | (proportion threatenedness)~latitude * ln(area) + (IUCN PA management category) | | | | |
| 4566.5 | (proportion threatenedness)~latitude*ln(area) | | | | |
| 4568.2 | (proportion threatenedness)~latitude*ln(area) + year | | | | |
| 4633.8 | (proportion threatenedness) ~ latitude:ln(area) + (IUCN PA management category) | | | | |
| 4634.1 | (proportion threatenedness) ~ ln(area) | | | | |
| 4637.4 | (proportion threatenedness) ~ latitude:ln(area) + ln(area) | | | | |
| 4637.4 | (proportion threatenedness) ~ (IUCN PA management category) | | | | |
| 4643.4 | (proportion threatenedness) ~ ln(area)*year | | | | |
| 4660.7 | (proportion threatenedness) ~ latitude:ln(area) | | | | |
| 4681.6 | (proportion threatenedness) ~ 1 [null model] | | | | |
| 4687.0 | (proportion threatenedness) ~ year | | | | |

**Supplementary Table 3.** Negative binomial generalized linear models examining how the total species richness in a protected area responds to its characteristics: its natural log-transformed area, its year of establishment, latitude of its centroid, its IUCN protected area management category, or the interaction between its year of establishment and its natural log-transformed area. Model statistics are only included for the top three models by AIC. P-values are marked as follows: <0.001, ***; <0.01, **; and <0.05, *. This analysis includes 168,894 protected areas.

| **AIC** | **Variables** | **Coefficients**  **Estimate Std. error z-val p-val** | **Deviance (degrees of freedom)** | **McFadden pseudo-r^2^** |
| --- | --- | --- | --- | --- |
| 1,127,863 | (species richness) ~ ln(area) * year + latitude + (IUCN PA management category) | intercept 3.61 0.009 380.36 <2.0·10^-16^***  ln(area) -6.03·10^-3^ 1.36·10^-3^ -4.44 <9.2·10^-6^***  est. year -1.83·10^-5^ 2.46·10^-6^ -7.47 <8.1·10^-14^***  latitude -3.33·10^-2^ 1.35·10^-4^ -246.69 <2.0·10^-16^***  IUCNcatIb 1.15·10^-1^ 1.19·10^-2^ 9.69 <2.0·10^-16^***  IUCNcatII 3.27·10^-1^ 9.88·10^-3^ 33.07 <2.0·10^-16^***  IUCNcatIII 8.55·10^-2^ 7.77·10^-3^ 11.00 <2.0·10^-16^***  IUCNcatIV 0.25 6.48·10^-3^ 39.03 <2.0·10^-16^***  IUCNcatV 0.60 6.83·10^-3^ 88.29 <2.0·10^-16^***  IUCNcatVI 0.18 8.91·10^-3^ 19.79 <2.0·10^-16^***  ln(area):year 7.93·10^-6^ 7.32·10^-7^ 10.82 <2.0·10^-16^*** | Null: 291,873 (174,766)  Residual: 179,279 (174,756) | 0.072 |
| 1,127,970 | (species richness) ~ ln(area):year + lat + (IUCN PA management category) | intercept 3.58 8.58·10^-3^ 417.16 <2.0·10^-16^***  latitude -3.33·10^-2^ 1.34·10^-4^ -249.14 <2.0·10^-16^***  IUCNcatIb 0.12 1.19·10^-2^ 9.90 <2.0·10^-16^***  IUCNcatII 0.32 9.88·10^-3^ 32.85 <2.0·10^-16^***  IUCNcatIII 8.87·10^-2^ 7.78·10^-3^ 11.40 <2.0·10^-16^***  IUCNcatIV 0.25 6.48·10^-3^ 39.28 <2.0·10^-16^***  IUCNcatV 0.62 6.60·10^-3^ 94.00 <2.0·10^-16^***  IUCNcatVI 0.18 8.91·10^-3^ 19.93 <2.0·10^-16^***  ln(area):year 4.95·10^-6^ 2.89·10^-7^ 17.12 <2.0·10^-16^*** | Null: 291,729 (174,766)  Residual: 179,304 (174,758) | 0.072 |
| 1,146,734 | (species richness) ~ ln(area):year + lat | intercept 3.96 5.93·10^-3^ 668.93 <2.0·10^-16^***  latitude -3.33·10^-2^ 1.32·10^-4^ -260.66 <2.0·10^-16^***  ln(area):year 5.82·10^-6^ 2.82·10^-7^ 20.67 <2.0·10^-16^*** | Null: 262,726 (174,766)  Residual: 179,768 (174,764) | 0.056 |
| 1,147,111 | (species richness) ~ ln(area) + latitude | | | |
| 1,147,116 | (species richness) ~ latitude | | | |
| 1,196,491 | (species richness) ~ ln(area) * year | | | |
| 1,196,514 | (species richness) ~ ln(area):year + year | | | |
| 1,197,810 | (species richness) ~ ln(area) + year | | | |
| 1,201,134 | (species richness) ~ ln(area):year + ln(area) | | | |
| 1,201,469 | (species richness) ~ ln(area):year | | | |
| 1,205,341 | (species richness) ~ ln(area) | | | |
| 1,215,043 | (species richness) ~ 1 [null model] | | | |

**Supplementary Figure 1. Country profile plots.**
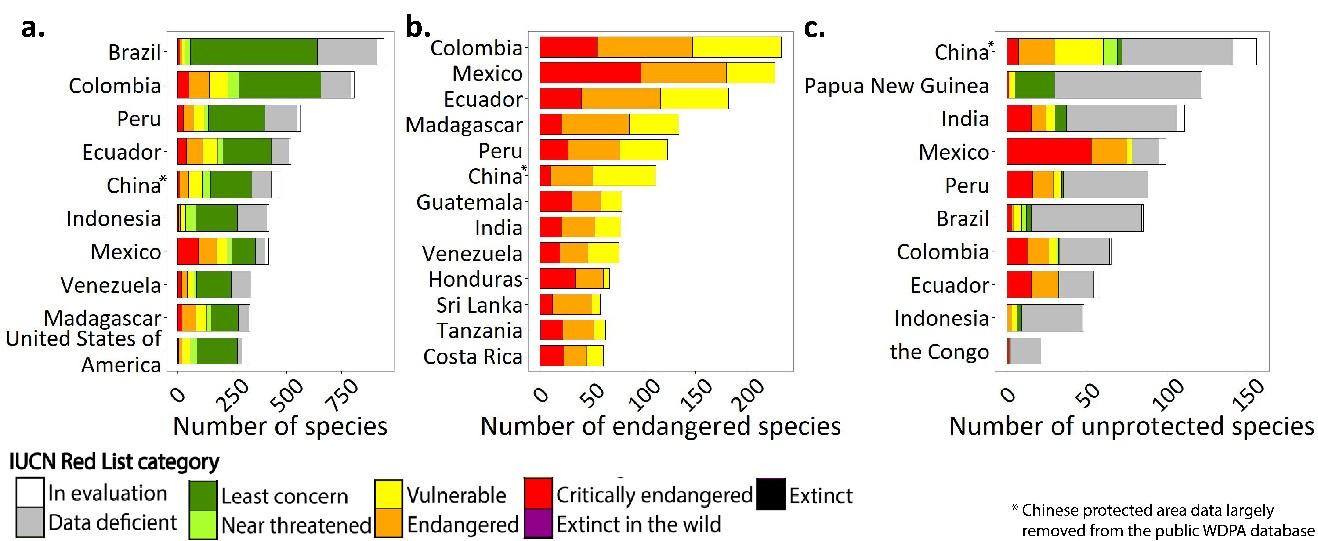


(a.) Top ten global countries in terms of total amphibian species richness, using all IUCN statuses. (b.) Top ten global countries in terms of threatened (VU, EN, CR, EW, EX) amphibian species, highlighting only IUCN threatened categories. (c.) Top ten countries in terms of amphibian species richness entirely unprotected by the current protected area network, showing all IUCN statuses.

Mexico has 1,171 protected areas currently registered in the World Database of Protected Areas (WDPA). 418 amphibian species occur within this country’s borders, and 294 of those species are endemic to Mexico. In our analysis, Mexico is seventh of countries with the highest amphibian richness (Fig. S1.A). 56% (232 species) of Mexico’s amphibian diversity is threatened with extinction (Fig. S2.B). Ninety-nine, or over 23%, of Mexican amphibian species currently show no overlap with the existing protected area network (Fig. S2.C). Adding only 7.15% (140,414 km2) of Mexico’s terrestrial area (1,962,939 km^2^) would provide coverage to all currently unprotected species, bringing Mexico’s total terrestrial PA coverage to over 32% allowing Mexico to reach its 30x30 goal.

**Supplementary Figure 2.** **Mexico: a case study.**
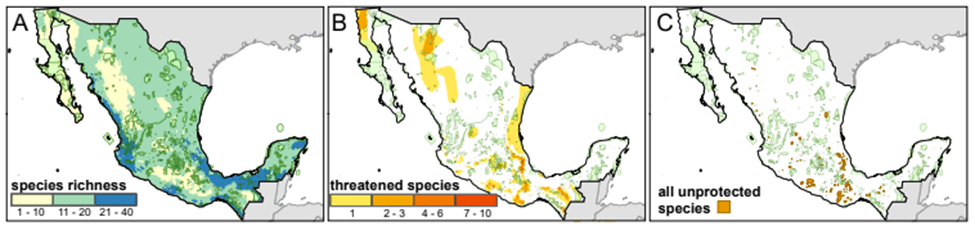


(a.) Amphibian alpha-richness relative to protected area placement (green outlined polygons). (b.) Threatened amphibian species richness relative to protected areas (green outlined polygons). (c.) The ranges of amphibian species with no overlap with the existing PA network (orange filled polygons) relative to protected area placement (green outlined polygons).
