# Supplementary material for: Microreserves are an important tool for amphibian conservation
Source: Commun Biol. 2024 Sep 19;7:1177. doi: 10.1038/s42003-024-06510-0 (PMC11413221; doi:10.1038/s42003-024-06510-0)
Supplement: Supplementary file 3 — Reporting summary [file 42003_2024_6510_MOESM3_ESM.pdf]

nature portfolio

Corresponding author(s): Emma Steigerwald
Julia Chen
Julianne Oshiro
Vance T. Vredenburg
Alessandro Catenazzi
Michelle S. Koo

Last updated by author(s): May 27, 2024

Reporting Summary

Nature Portfolio wishes to improve the reproducibility of the work that we publish. This form provides structure for consistency and transparency in reporting. For further information on Nature Portfolio policies, see our Editorial Policies and the Editorial Policy Checklist.

Statistics

For all statistical analyses, confirm that the following items are present in the figure legend, table legend, main text, or Methods section.

n/a	Confirmed	
☐	☒	The exact sample size (n) for each experimental group/condition, given as a discrete number and unit of measurement
☐	☒	A statement on whether measurements were taken from distinct samples or whether the same sample was measured repeatedly
☐	☒	The statistical test(s) used AND whether they are one- or two-sided<br>*Only common tests should be described solely by name; describe more complex techniques in the Methods section.*
☐	☒	A description of all covariates tested
☐	☒	A description of any assumptions or corrections, such as tests of normality and adjustment for multiple comparisons
☐	☒	A full description of the statistical parameters including central tendency (e.g. means) or other basic estimates (e.g. regression coefficient) AND variation (e.g. standard deviation) or associated estimates of uncertainty (e.g. confidence intervals)
☐	☒	For null hypothesis testing, the test statistic (e.g. F, t, r) with confidence intervals, effect sizes, degrees of freedom and P value noted<br>*Give P values as exact values whenever suitable.*
☒	☐	For Bayesian analysis, information on the choice of priors and Markov chain Monte Carlo settings
☒	☐	For hierarchical and complex designs, identification of the appropriate level for tests and full reporting of outcomes
☐	☒	Estimates of effect sizes (e.g. Cohen's d, Pearson's r), indicating how they were calculated

Our web collection on statistics for biologists contains articles on many of the points above.

Software and code

Policy information about availability of computer code

Data collection	Data was downloaded from several online repositories manually, as described under "Data acquisition" in our Methods.
Data analysis	All data analysis and visualization was performed in Quantum GIS v3.2, ESRI ArcGIS v10.8, and in R v4.1.1 using libraries stringr v1.4.0, dplyr v1.0.7, plyr v1.8.6, tidyr v1.1.3, lessR v4.1.4, forcats v0.5.1, data.table v1.14.2, hexbin v1.28.2, ggridges v0.5.3, ggplot2 v3.3.5, raster v3.4., scales v1.1.1, nortest v1.0-4, MASS v7.3-54, pscl v1.5.9, car v3.0-11, and cowplot v1.1.1.

For manuscripts utilizing custom algorithms or software that are central to the research but not yet described in published literature, software must be made available to editors and reviewers. We strongly encourage code deposition in a community repository (e.g. GitHub). See the Nature Portfolio guidelines for submitting code & software for further information.

Data

Policy information about availability of data

All manuscripts must include a data availability statement. This statement should provide the following information, where applicable:

- Accession codes, unique identifiers, or web links for publicly available datasets
- A description of any restrictions on data availability
- For clinical datasets or third party data, please ensure that the statement adheres to our policy

Data used in our analysis have been uploaded to DataDryad (DOI: will be provided upon publication).

Research involving human participants, their data, or biological material

Policy information about studies with human participants or human data. See also policy information about sex, gender (identity/presentation), and sexual orientation and race, ethnicity and racism.

Reporting on sex and gender	NA
Reporting on race, ethnicity, or other socially relevant groupings	NA
Population characteristics	NA
Recruitment	NA
Ethics oversight	NA

Note that full information on the approval of the study protocol must also be provided in the manuscript.

Field-specific reporting

Please select the one below that is the best fit for your research. If you are not sure, read the appropriate sections before making your selection.

☐ Life sciences ☐ Behavioural & social sciences ☒ Ecological, evolutionary & environmental sciences

For a reference copy of the document with all sections, see nature.com/documents/nr-reporting-summary-flat.pdf

Ecological, evolutionary & environmental sciences study design

All studies must disclose on these points even when the disclosure is negative.

Study description	We use publicly-availably data to do a number of tests and data visualizations, organized around the idea that some taxa will require the targeted establishment of small protected areas if we are to substantially represent them in the growing protected area estate. We use global amphibian diversity as our featured example. We start with simple Kruskal-Wallis and Wilcoxon rank sum tests to demonstrate that amphibian distributional ranges are smaller than those of other terrestrial vertebrates. We then summarize updated, expert-curated data to report the current coverage of amphibian diversity by global protected areas. We visualize how the coverage of amphibian diversity by the PA network has grown over the last century and a half, then use generalized linear models to explore how first the total species richness in a PA and then the proportion of species in a PA that are threatened responds to the age of that PA, its size, the latitude of its centroid, and its IUCN category. We resample the global PA network with replacement 1000 times to examine how the number of amphibian species protected by it grows through the addition of different sized protected areas. Finally, we visualize data at a regional level, looking at how different global areas are characterized by different distributions of protected area sizes and differential coverage of their amphibian diversity.
Research sample	This research focuses on global amphibian species, as amphibians are undergoing global declines and extinctions, with habitat loss serving as a major driver, yet are highly unlikely to be the focal taxa of new PAs. Amphibians' small ranges and high rates of endemism makes them good candidates for demonstrating how the assumption that big protected areas are best for protecting biodiversity will neglect to protect many species with small, disjunct distributions. The source for the existing datasets we used for these analyses are disclosed under "Data collection", below.
Sampling strategy	The sample size for our analyses were determined by the total sample size of the existing datasets we used (as described under 'Data collection', below), with the small exclusions remarked upon (under 'Data exclusions', below).
Data collection	We used amphibian range maps from AmphibiaWeb and IUCN (available for 7,094 species–over 83% of named amphibian species). For mammals and reptiles, we used ranges from the IUCN and the Global Assessment of Reptile Distributions group (10,811 reptiles and 5,850 mammals). For birds, we used ranges for 10,487 species from BirdLife International that excluded species they consider sensitive and joined all range polygons for each species, as they were originally separated into 'resident', 'breeding season', 'non-breeding season', 'passage', and 'seasonal occurrence uncertain' components. We acquired species' conservation status from the

	IUCN Red List of Threatened Species. For amphibians, we included expert-curated provisional statuses. We used the 240,999 PA polygons in terrestrial biomes from the World Database of Protected Areas (WDPA) database.
Timing and spatial scale	The study is global in scope, to give a report of the status of amphibian conservation globally. For most analyses, we use a single time point-- data updated to the present. However, we do include some analyses including the date of protected area establishment, as declared by the World Database of Protected Areas, which includes dates as early as 1800.
Data exclusions	In the WDPA dataset, we trimmed away any portions of protected areas that overlapped marine habitats, as there are no marine amphibians. The Russian Federation, Estonia, Saint Helena, Ascension, Tristan da Cunha, and China withhold all or part of their PA spatial data from public release3, and we do not make assumptions about the current status of PAs previously reported to the WDPA and later withdrawn. Polygons of PAs that overlapped with each other were merged. We removed two polygons by searching for records that included the text "not protected", "degazetted," "proposed," "recommended," "in preparation," or "unset". We do not filter out WDPA based on their designated IUCN category in our analyses, excepting in our generalized linear models, since IUCN category was a factor in those analyses. It should be noted that for all analyses in which area of PAs is used, we use PA terrestrial area as reported by the WDPA (PA area less its marine area, i.e. GIS_AREA - GIS_M_AREA).
Reproducibility	Since the analysis of how amphibian coverage grows within a PA network based on the addition of different sizes of PAs depended on resampling of the global database of protected areas, we repeated the resampling with 1000 iterations.
Randomization	Samples were not randomly assigned to groups for any analyses in this study.
Blinding	Blinding was not relevant to this study, as it did not involved direct observation or measurement but rather pre-published datasets.

Did the study involve field work? ☐ Yes ☒ No

Reporting for specific materials, systems and methods

We require information from authors about some types of materials, experimental systems and methods used in many studies. Here, indicate whether each material, system or method listed is relevant to your study. If you are not sure if a list item applies to your research, read the appropriate section before selecting a response.

Materials & experimental systems

n/a	Involved in the study
☒	☐ Antibodies
☒	☐ Eukaryotic cell lines
☒	☐ Palaeontology and archaeology
☒	☐ Animals and other organisms
☒	☐ Clinical data
☒	☐ Dual use research of concern
☒	☐ Plants

Methods

n/a	Involved in the study
☒	☐ ChIP-seq
☒	☐ Flow cytometry
☒	☐ MRI-based neuroimaging

Plants

Seed stocks	NA
Novel plant genotypes	NA
Authentication	NA
