# Supplementary material for: Microreserves are an important tool for amphibian conservation
Source: Commun Biol. 2024 Sep 19;7:1177. doi: 10.1038/s42003-024-06510-0 (PMC11413221; doi:10.1038/s42003-024-06510-0)
Supplement: Supplementary file 4 — Spanish abstract [file 42003_2024_6510_MOESM4_ESM.docx]

Las microrreservas son una herramienta importante para la conservación de los anfibios

Las iniciativas para proteger el 30% de la tierra de aquí a 2030 impulsan la evaluación de cómo subsanar eficazmente las deficiencias de la red mundial de áreas protegidas (PA en este artículo). Centrándonos en los anfibios, la clase de vertebrados más vulnerable, ilustramos el valor de conservación de las "microreservas", término que empleamos aquí para referirnos a las reservas de <10 km^2^. Informamos de que la red sigue infrarrepresentando a los anfibios amenazados y que, a pesar de esta clara deficiencia en la conservación terrestre, la creación de PA que protegen a los anfibios se ralentizó después de 2010. Demostrando algo previamente asumido -que los anfibios generalmente tienen áreas de distribución más pequeñas que otros vertebrados terrestres- demostramos que las microrreservas podrían proteger una porción sustancial de muchas áreas de distribución de anfibios, particularmente especies amenazadas. Descubrimos que las microrreservas existentes son capaces de albergar una riqueza de especies de anfibios similar a la de PA entre 1.000 y 10.000 veces más grandes, y demostramos que la alta diversidad beta de los anfibios significa que las microrreservas añadidas a una red de PA en crecimiento cubren las especies de anfibios 1,5-6 veces más rápido que las categorías de PA de mayor tamaño. Proponemos que para frenar la pérdida global de biodiversidad es necesario considerar seriamente el potencial de conservación de las microrreservas, utilizándolas para capturar especies endémicas con distribuciones pequeñas que, de otro modo, podrían omitirse por completo de la red de PA.
